# Antibiotics in Catalan Primary Care: Prescription, Use and Remedies for a Crisis of Care

**DOI:** 10.1080/01459740.2023.2256451

**Published:** 2023-09-25

**Authors:** Adam Brisley, Helen Lambert, Carla Rodrigues

**Affiliations:** a Business and Technology Department, La Salle, Universitat Ramon Llull, Barcelona, Spain; b Population Health Sciences, Bristol Medical School, University of Bristol, Bristol, UK; c Department of Sociology, University of Amsterdam, Amsterdam, The Netherlands

**Keywords:** Antibiotics, antimicrobial resistance, care crisis, Catalonia, primary care, Spain

## Abstract

Antimicrobial resistance is one of the twenty-first century’s major health challenges. Linked to the extensive use of antibiotics and other antimicrobials, resistance occurs when microbes stop responding to medications. Rates of antibiotic consumption in Spain are among the highest in Europe. Drawing on research conducted in Catalonia, in this article we present findings from ethnographic fieldwork and semi-structured interviews with general practitioners, residents of Barcelona, and professionals who have worked in antibiotic stewardship. We argue that the circulation of antibiotics should be understood in relation to broader historical processes and the deficient systems of health and social care provision they have produced.

## RESUM

Relacionada amb l’ús extensiu d’antibiòtics i altres antimicrobians, la resistència als antimicrobians és un dels principals reptes de la salut del segle XXI. Les taxes de consum d’antibiòtics a Espanya es troben entre les més altes d’Europa. A partir de la recerca realitzat a la comunitat autònoma de Catalunya, aquest article informa de les conclusions del treball de camp etnogràfic i de les entrevistes semiestructurades amb metges, amb professionals que han treballat en política i recerca d’antibiòtics i amb residents de Barcelona. Defensem que la circulació d’antibiòtics s’ha d’entendre en relació amb els processos històrics més amplis i els sistemes deficients d’atenció sanitària i social que s’han produït al llarg d’aquests.

Antimicrobial resistance (AMR or drug-resistant infection) is one of the twenty-first century’s major health challenges. Linked to the extensive use of antimicrobials in medicine, food production and other industrial activities, microbes adapt as a result of exposure to antimicrobials and become “resistant” (unresponsive) to these medications, making infections more difficult to treat and increasing the risk of severe illness and death (WHO 2021). An estimated 4.95 million deaths were associated with bacterial AMR in 2019 (Murray et al. 2022). However, AMR has probably been accelerated by the COVID-19 pandemic. Many patients admitted to hospital with COVID-19 received antibiotics and antimicrobial stewardship activities were largely suspended due to staffing pressures on overstretched health systems (Cong et al. 2021; Hsu 2020).

Fears about the return of a pre-antibiotic age have prompted the World Health Organization (WHO) and many national governments to adopt action plans aimed at increasing awareness, reducing infections and “optimizing” the use of antimicrobials (WHO 2015). Spain published its first national action plan in 2014 (AEMPS 2014; see also AEMPS 2022), but critics have since bemoaned the lack of concrete action by policymakers, highlighting gaps in antibiotic prescribing data and failure to tackle illegal over-the-counter sales (Belomote 2016; Llor 2017). Rates of antibiotic consumption in Spain remain among the highest in Europe. As in other countries, primary care accounts for the bulk of human antibiotic consumption via pharmacies, dentists, and general practitioners (GPs) (ECDC 2019). The Catalan government, which is responsible for health care in the autonomous community of Catalonia, has adopted a strategy to optimize the prescription and use of antibiotics in accordance with the Spanish action plan (GenCat 2021). Research conducted in the region suggests Catalonia faces similar problems to the rest of Spain (Llor and Cots 2009).

Over the past decade, numerous studies have sought to understand variations between regional rates of antibiotic consumption by exploring the attitudes of primary care physicians regarding antibiotic use (Lopez-Vazquez et al. 2012; Md Rezal et al. 2015; Rodrigues et al. 2013). Patient safety concerns (that patients may go on to develop serious illness if antibiotics are not prescribed) are frequently cited as influencing prescribing in primary care in Spain. Other commonly reported factors include a lack of knowledge about appropriate prescription and acquiescence with patient requests for antibiotics (Gonzalez-Gonzalez et al. 2015; Vazquez-Lago et al. 2011). Research into antibiotic use often takes a rather normative approach (Rodrigues 2020; Willis and Chandler 2019), assuming that high rates of antibiotic prescribing indicate inappropriate or even “irrational” (GenCat 2021; Liu et al. 2019) decision-making. But framing antibiotic prescription as a problem of individual behavior obscures structural dimensions of prescribing associated with systems of health and social care provision (Chen et al. 2020; Michele de Oliveira et al. 2019; Willis and Chandler 2019). As recent discussions in medical anthropology and sociology have emphasized, understanding the use of antibiotics and other medicine requires interrogating what constitutes rationality in situated circumstances and how this might depend on, among other things, material constraints (Lambert et al. 2019; Rodrigues 2020).

In this article, we explore the prescription and use of antibiotics in Catalonia from the perspective of GPs, residents of Barcelona, and professionals working on antibiotic stewardship. We situate antibiotic consumption within its local socio-political and historical context and explore four key themes that our informants identified as problematic in the context of current attempts to reduce levels of antibiotic consumption. In so doing, we move the focus away from the attitudes of individual prescribers and users and toward more diffuse forms of social responsibility.

## Situating the prescription and use of antibiotics

In a review of 35 qualitative studies on primary care antibiotic prescribing, 12 addressed the link between prescribing and work pressure associated with patient volume, with only one not reporting any relationship between this factor and prescription (Rodrigues et al. 2013). Yet even when structural and systemic factors are included in analyses aiming to identify aspects for potential change and improvement, the attitudes of prescribers are often highlighted. In a study of primary care prescribers in Spain, for example, focus group participants described various “external factors” including availability of over-the-counter antibiotics, pharmaceutical industry influence, and health systems issues (Vazquez-Lago et al. 2011), but the only implications highlighted for practice and research were “fear of complications, complacency vis-à-vis patient pressure, and insufficient knowledge” (2011:6).

Physicians’ attitudes are so well researched in part because, like those of patients, they are viewed as potentially modifiable and therefore amenable to the kinds of knowledge-based interventions that currently predominate in global heath (e.g., WHO 2015; see also Will 2018; Charani and Holmes 2019; Lambert et al. 2019). This may foster an assumption that prescribers are the most important causal agents for reducing consumption of antibiotics, even while doctors themselves highlight a range of issues beyond their control. Recent ethnographic studies of antibiotic prescription and self-medication have challenged the notion that the use of antibiotics when not clinically necessary can be explained by the putatively irrational beliefs and attitudes of individual doctors and patients (Michele de Oliveira et al. 2019; Rodrigues 2020; Willis and Chandler 2019). Scholars have highlighted how clinicians working in health care settings in the global south often prescribe antibiotics to treat infections that result from unsanitary conditions, as well as to prevent such infections (Rajyowijati and Haak 2003; Willis and Chandler 2019). When treating infections, “clinicians often err on the side of over-treatment” (Charani and Holmes 2019:2) and not complying with the expectations of antibiotic stewardship programs is “not always tantamount to poor treatment of the patient” (2019:2–3).

Broom et al. (2014) describe hospital doctors’ antibiotic prescribing in Australia as guided by the desire to protect patients from clinical risks while managing time pressures. Doing (and being seen to do) everything possible for their patients disciplined doctors into “habitual practices” that did not “necessarily correlate with therapeutic guidelines or current best practice” (2014:87, see also Cabral et al. 2015). Likewise, ethnographic research on primary care antibiotic prescribing in China has explored the conflicting moral obligations that rural doctors feel toward their patients, with whom they wish to maintain good relations, their businesses, which depend on these relationships, and their duty to follow government guidance on antibiotic prescribing (Chen et al. 2020; Lambert et al. 2019). These accounts reveal that rationales for antibiotic prescribing are multiple, contingent, and often deeply embedded within the situated circumstances and socio-economic dynamics at play within particular local health care contexts (Rodrigues 2020).

Medical anthropology has long emphasized the need to consider the relationship between health system deficiencies and historic processes of structural violence (Farmer 1996). This means accounting for how economic pressures shape material realities of illness, care and (perceived) needs for medication (Biehl 2007). We argue that Spain’s and Catalonia’s socio-economic history shapes the situated circumstances in which antibiotic prescription and use occur.

## Spain’s care crisis

For much of the twentieth century, social welfare in Spain was characterized by limited state provision of services, intrafamilial pooling of resources and heavy dependence on women’s unpaid domestic labor (Miguélez and Recio 2010). Medical care was accessible via a patchwork of charities, private clinics for the wealthy, and an obligatory health insurance scheme called *Seguro Obligatorio de Enfermedad* (SOE). Funded by compulsory employer and worker contributions, SOE was restricted to low-income workers and excluded some industries (Perdiguero-Gil and Comelles 2019).

Democratic reforms introduced after the dictator Franco’s death created a ministry of health and extended the right to access publicly funded health care to the entire Spanish population. The 1978 constitution granted regional autonomy to Catalonia and devolved responsibility for health care the new Catalan government (Gobierno de España 2008). But Spain’s post-Franco period of welfare consolidation did not last long. In the 1990s, cuts to welfare expenditure were introduced along with a series of labor market reforms, including new temporary contracts with reduced social security, severance, and redundancy entitlements. By the end of the decade, the Spanish workforce was more casualized than its Northern European counterparts, with the worst effects of deregulation concentrated on new entrants to the job market, mainly young people, migrants, and women (McVeigh 2005).

Leading up to the 2008 global financial crash, Spain appeared to be performing well in economic terms, enjoying average annual growth of 3.7% from 1995–2007 (Buendía and Molero-Simarro 2018). This ostensibly rosy economic picture hid the fact that even before the crash, Spain’s care sector was already beset by a crisis resulting from declining levels of public investment, an underdeveloped social care system, and the increasing care needs of an aging population (Pérez-Orozco 2006; Buendía and Molero-Simarro 2018; Campillo-Poza 2018).

The financial crisis in Spain resulted in the dramatic collapse of the housing market, a six-year recession and unemployment reaching 27%. In the following decade, Spanish and Catalan governments responded to rising levels of public debt by introducing programs of fiscal austerity and labor market reform. The weakening of worker protections and restructuring of unemployment benefit resulted in reduced salaries and increased casualization. These policies affected the health and social care sector both directly through major reductions in public spending (Campillo-Poza 2018) and indirectly through the adverse effects of increasing socio-economic inequality and insecurity on people’s health and wellbeing (Cervero-Liceras et al. 2015; Gili et al. 2013). A host of policies and programs that were intended to address deficits in the Catalan and Spanish social care system – including an early education and care service and a long-term care system to address the needs of the aging population – were either reversed or abandoned (Campillo-Poza 2018).

What resulted has been described as a “crisis of care” (Bofill-Poch 2018; Campillo-Poza 2018, see also Pérez-Orozco 2006; Fraser 2016), wherein pressure from several directions squeezes a “key set of social capacities: those available for birthing and raising children, caring for friends and family members, maintaining households and broader communities, and sustaining connections more generally” (Fraser 2016:99). In this article, we draw on the accounts of GPs, residents of Barcelona, and others to explore the relationship between this crisis, the broader historical, social, and political processes producing it, and the use of antibiotics in Catalan primary care.

## Methods and setting

The article is based on six months fieldwork conducted by the first author in Barcelona between 2018 and 2019. Drawing on existing local contacts and a snowball approach to participant recruitment, the first author sought to record a broad range of opinions and perspectives relating to AMR and antibiotic prescription and use in this setting. In addition to gathering and reviewing health service policy documents and numerous informal conversations with residents of Barcelona, this approach resulted in 13 in-depth, semi-structured interviews with 10 participants, shown in Table 1.

**Table 1.** Research participants.

Identity code	Category	Gender and Age
GP1	General practitioners	M, 55
GP2		M, 47
GP3		F, 53
GP4		F, 47
Nu1	Primary care nurse	F, 37
CR1	Clinical researcher	M, 54
Ph1	Director of pharmacy	F, 57
Re1	Residents	M, 34
Re2		F, 36
Re3		F, 34

An ethnographic approach to interviews was used, with gatekeepers treated as key informants and asked not only to describe personal experiences but encouraged to reflect on their professional practice and provide intercultural interpretation and historical context (for this approach in relation to ethnography see Hockey 2002; O’Reilly 2009:132–137; Spradley 1979). Analysis was conducted throughout data collection in an iterative, reflexive style, with each new interview or conversation informing the next (O’Reilly 2009). Interviews were conducted in English and Spanish.

## Prescribing and consuming antibiotics in Catalonia

In this section we present our main findings, beginning by drawing on existing literature and ethnographic materials to situate recent antibiotic policy in historical context. The subsequent four subsections explore issues that our informants identified as problematic in relation to attempts to reduce antibiotic consumption, namely, a lack of consistent policy focus, systemic pressures on primary care services, patient requests for antibiotics and over-the-counter sales of antibiotics without prescription. In the final section of the article, we explore the relationship between these issues, Catalonia’s crisis of care and the broader political economic forces that are producing it.

### Antibiotics and regulatory regimes

The first decades of antibiotics in Spain were characterized by strict control of state supplies on the one hand, and abundant availability of black-market antibiotics on the other (Santesmases 2018). Widespread tuberculosis resulting from poor living conditions and basic sanitation plagued Spain during the nineteenth and early twentieth century (Molero-Mesa 1998). Spain emerged from its civil war of the 1930s as a fascist/corporatist state committed to the economic doctrine of autarky. Known as the “years of hunger,” the first decades of the dictatorship were defined by scarcity, depopulation, infection, and the terror imposed by the Franco regime. Liberal economic reforms in the 1950s partially opened the Spanish economy to external investment, bringing an end to the early regime’s more isolationist policies (Santesmases 2018). But despite the expansion of industrial centers like Madrid, Barcelona, and Valencia in the 1950s and 1960s, public services remained severely underdeveloped (McVeigh 2005; Miguélez and Recio 2010). As Santesmases (2018) history of penicillin in Spain illustrates, these conditions helped to ensure a heavy reliance on antibiotics as effectively a treatment for poverty as well as infection throughout Spain for much of the twentieth century.

High levels of pharmaceutical consumption were exacerbated by the SOE health insurance scheme, which placed drugs rather than services at the center of health care. SOE facilities were scarce and under-resourced. Compulsory contributions meant patients sought to recover their money by obtaining prescription medicines, which were free to scheme members. With little time to examine patients, doctors in turn issued prescriptions to avoid dissatisfaction (Perdiguero-Gil and Comelles 2019). Regulations and prescription requirements for antibiotics were gradually introduced from the 1950s onward, but these measures were intended to control supply rather than address issues associated with consumption. The informal circulation of antibiotics remained largely uncontrolled throughout the dictatorship.

In the 1980s, with the post-Franco expansion of the national health system, antimicrobial resistance and antibiotic consumption began to be addressed at the policy level (Santesmases 2018). Ph1, a 57-year-old director of pharmacy in the Catalan health service, began her career during this period. She spoke about a tradition found throughout Spain in her childhood of “using antibiotics for everything” and remembered her parents casually giving her antibiotics when she had a cold or sore throat, telling her, “take this, it’s good.” By the 1980’s, she said, Spain had a “big problem with antibiotics” and was gaining an international reputation for high levels of AMR.

Ph1’s work involved approving new drugs as they became available in Catalonia and providing guidance and training for primary care prescribers. She remembered working on antibiotic use and said that significant progress was made during that period, but this early focus did not last long:

Ph1: So, at that time we worked quite intensively … with the problem of excessive use here in Catalonia with antibiotics. After we forgot absolutely the antibiotics because we have chronic treatments that absorb our attention absolutely, and now we turn again [to] antibiotics.

Ph1 explained that during the 1980s and 1990s the market for chronic illness treatments, particularly anti-depressive and anti-psychotic medicines, rapidly increased. She and her colleagues were obliged to work on these new drugs as they became available, leaving little time for the management of antibiotics.

GP1 recalled that when he began work in Tarragona in 1992, rates of AMR in Spain were among the highest in the world and yet he found that few people were “interested in the management of infectious diseases in primary care.” The only available guidance for the prescription of antibiotics came from secondary care, but while hospital doctors knew “a lot about the infections they see,” they understood less about the infections that frequently occurred in general practice. GP1 organized a study group with fellow GPs and began work on a book of recommendations for the primary care treatment of infections, which was later published by the Catalan Society of Family Medicine.

Prescription guidelines have since become standard in Catalan primary care. There have also been periodic public health campaigns and locally successful interventions to reduce prescribing through professional training and discussion (Belomote 2016; Molero et al. 2019). Moreover, all the GP informants commented favorably on a point of care test for the detection of *A streptococci* bacteria, or “strep-A” test, being made available in Catalonia. However, our informants on the whole were critical of the slow pace of change and the lack of action that had resulted from the 2014 Spanish national action plan.

### Changing prescription practices

Two clinician informants understood high rates of antibiotic prescription as driven by outdated ideas and practices. GP2 explained how patterns of antibiotic prescription and use reflected a “Spanish tradition” of trying to “solve everything with a pill.” He said that there was a widespread convention among many GPs of prescribing azithromycin 600 mg once a day for three days for the treatment of common colds. This was apparently based on what he called a “medical myth” – held by doctors and patients alike – that if left untreated, the cold virus could travel from the sinuses down through the esophagus into the lungs, causing pneumonia.

Similarly, GP1 said that prescription practices reflected the “backgrounds” of physicians and said that “one of the toughest things” was “trying to change the GPs’ minds.” Young physicians emulated the customs of older generations of physicians who had routinely used antibiotics in ways that he now considered to be inappropriate: “if you see that a senior doctor prescribes antimicrobial for an infection that is mainly viral[…], you are going to continue with this practice.”

Three GP informants described recent changes in the attitudes of their colleagues and patients toward antibiotics. For instance, while describing his own practices, GP2 said that previously he would prescribe antibiotics based on “simple clinical criteria” such as the coloration of the sputum, with a greener color indicating the need to prescribe. But nowadays he was more cautious:

GP2: [In the past] A cough that is lasting for two months probably … we’ll need to treat it with *amoxicilina*. [But] perhaps [today] it’s not like this, now I talk to my patients and I say “well perhaps during all the winter there will be cold, there will be humidity, and you will have cough and you will have to drink more water, keep warm, and all that,” and I don’t give them antibiotics. But I think [] if you review my prescribing, a great cut could be seen from two years ago till now.

Yet while our informants spoke about their colleagues’ changing attitudes, they also highlighted several interrelated problems. The first was that antibiotic stewardship was not being prioritized sufficiently at the policy level. CL1, a former GP and clinical researcher, described the implementation of the 2014 national strategy as “timid” and joked that the problem of AMR had got so bad in Spain that even “the ministry has started to do something.” He said that while AMR should be in the “top ten” priorities in health care, it was not seen as such by Catalan or Spanish policymakers. GP1 characterized the existing approach as “progressing very slowly” and suggested that rather than reflecting genuine concern for the problem of AMR, the 2014 policy was in part intended to placate the demands of international stakeholders:

GP1: I think, first of all from the Spanish perspective, I think antimicrobial resistance isn’t on the agenda of politicians in Spain – neither in Catalonia. But from the Spanish perspective – this is what I see and this is something that we discuss with other colleagues – I think the Spanish Minister of Health is obliged to follow some guidelines from Europe at the European level – the problem of antimicrobial resistance is on the agenda – fortunately, and I think the Spanish government is obliged to do something.

Ph1 concluded that whilst the question of AMR and antibiotics had never been abandoned in Catalonia, neither had it ever become a main focus of policy because of more pressing and immediate priorities. These informants’ accounts suggest that AMR may continue to struggle for attention in policy and governance.

### Systemic pressures and chronic illnesses

A second problem our informants identified concerned systemic pressures on the Catalan health system. GP1 observed that some colleagues did not use technologies like the Strep-A rapid diagnostic test during busy clinics simply because it takes time:

GP1: … it’s amazing, but for instance in my center, if I have to proceed with the strep-A procedure, I have to leave the surgery, the consultation, I have to go to another office, because we have the strep-A centralized in a cupboard at the entrance … and it takes time. I have to go to the other office to take the strep A, to come back to my office and proceed with the technique – it takes time. You’re not going to believe it, but in my opinion, this is the main limitation of the technique from my point of view.

Likewise, GP3 said that doctors prescribe because it is easier than taking the time to explain things to patients during busy clinics. Moreover, she said, some GPs prescribe antibiotics even when the test is negative:

GP3: A lot of colleagues now, they do a prescription of antibiotics when the people say “I want an antibiotic” for example. In my case, for example, my colleagues, we have Strep-A in our consultations, and a lot of them don’t use it yet – sometimes when they use it and it is negative, they continue prescribing an antibiotic …

When asked the reasons for this, GP3 explained:

GP3: Because I think it’s easier to prescribe an antibiotic than to explain to the people what the problem is, this is a virus – it’s quicker, it’s quicker to do a prescription than explain all of this.

GP1 explained why time was so limited in primary care. He said that in theory, nine minutes of consultation time was allocated to each patient on his daily list. In practice, however, consultations were much shorter because he also had to allocate time for patients in urgent need of care who arrived without an appointment – a situation which was sometimes so common in the winter months that it would effectively double the number of patients he saw. In consequence he generally had about five minutes per patient.

In addition, patients would also attend the clinic purely for “bureaucratic red tape things” such as sicknotes or having forms signed. These “bureaucratic patients,” according to GP1, accounted for about a third of his consultations. He lamented the absence of a patient self-certification system such as exists in other countries, wherein a doctor’s signature on a sicknote is only mandatory after the first two weeks of absence from work. He said that, across Spain, employers generally did not trust their employees and therefore expect medical certification before allowing them to miss work. GP1 contrasted “bureaucratic patients” with those attending for medical reasons. Of these, he said, the majority suffered from mental health or chronic conditions or both:

GP1: … most of the patients have chronic diseases – I think we are overwhelmed, and this is I think the correct word for that, we are overwhelmed by repeated visits of patients with chronic diseases.

The burden of chronic disease patients was mentioned by several other informants. Nu1, a primary care nurse, said that the vast majority of her work was now taken up managing the care needs of patients over 70 years old who suffered from chronic musculoskeletal illnesses. CL1 said that the management of chronic conditions was the greatest challenge facing the Catalan health system and associated it with the problem of demographic aging. GP2 described “demand” as driven by patients with complex chronic needs and mental health problems, creating pressure that was exacerbated by systemic resource shortages originating from the high cost of cancer medicines among other factors. Ph1 described a similar problem:

Ph1: … it’s a problem everywhere that you have increasing number of people that have chronic conditions … I know that the waiting lists – a very big problem … the hospitals have collapsed; primary care [practitioners] say that they cannot afford all these people that come to the visits. So, there is a problem of pressure, existential pressure – very, very high, and the resources probably aren’t enough.

GP4 and GP1 also discussed a certain group of patients who “consumed a lot of medications.” GP1 referred to them as “hyper-frequency patients,” or patients who would “repeatedly, repeatedly, repeatedly ask for appointments.”

Our informants’ accounts of systemic pressures are corroborated in the literature. Thirty-eight percent of the adult population of Catalonia has one or more chronic illnesses, a problem that is associated with Catalonia’s aging population as well as the rising levels of marginality and socioeconomic inequality that have been experienced in the territory in recent years (Departament de Salut 2021). In addition to elderly people, women and people with lower incomes are more likely to suffer from one or more chronic illness (Coronado-Vázquez et al. 2019). The “hyper-frequency patient” and “complex chronic patient” mentioned by some informants refer to this broader trend in which health care need, use, and spending tend to concentrate around people with multiple chronic co-morbidities (Iglesias et al. 2021; Vela et al. 2017). As GP1 noted, “hyper-frequency patients” also tend to live alone, have little social and family support, and are from communities experiencing significant socio-economic deprivation.

### “Patient demand” for antibiotics

In addition to pressures on primary care described above, which limited doctors’ opportunities to modify their antibiotic prescribing practices, patients were perceived to continually demand antibiotics. GP1 said that while his patients had more frequently requested antibiotics in the early years of his career, he was nevertheless still asked to prescribe antibiotics “every day.” Likewise, GP3 and GP4 said that patients frequently asked for antibiotics by name. “Patient demand” for antibiotics is a commonly reported research finding (Gonzalez-Gonzalez et al. 2015; Md Rezal et al. 2015). As others have argued, however, while prescribers and health researchers often refer to “Patient demand” as an independent factor, the desire for medicines may be better understood as relational (Britten 2008; Rodrigues 2020). Prescribing has long been understood by social scientists as a social exchange that goes beyond strictly medical purposes (Hall 1980; Pellegrino 1976; Stevenson et al. 2002), often not only meeting patients’ needs but also reaffirming doctors’ authority and expertise, even in the face of diagnostic uncertainty; “the repeated argument that the over-prescription of antibiotics is mainly driven by ‘patient demand’ needs to be further deconstructed and analyzed in concrete contextual circumstances” (Rodrigues 2020:9).

GPs in this study sometimes recognized the relational dimension of patient demand and described how patient expectations regarding antibiotics had been co-constituted over the years between doctors and patients. Thus GP2 attributed his patients’ frequent demand for antibiotics to the same “Spanish tradition” of trying to “solve everything with a pill” and felt patients had grown accustomed to taking antibiotics for viral infections because their doctors regularly prescribed in this way. Many of his patients continued to expect medical prescriptions when they became ill with colds or flu. Similar notions were echoed in many casual conversations between the first author and interlocutors in Barcelona. Re1, a post-doctoral researcher in her thirties had grown up in the city but now lived abroad for much of the year. She related high levels of antibiotic consumption to Catalonia’s socio-political history, explaining that under Franco, antibiotics had been “pushed onto” her mother’s generation for just about everything. She explained that whenever her mother was ill she still expected to receive antibiotics from her GP but nowadays rarely did, so instead she would buy them from a pharmacist with whom she was friendly and stockpile them at home.

Demand for antibiotics was not exclusively associated with an older generation, however. GP1 and GP3 said that younger patients understood more about AMR and were more receptive when told about the need to be prudent with antibiotic use, but neither GP said that patients of any particular age were more likely to ask for antibiotics. When asked directly which patients were likely to seek antibiotics, GP2 talked about young people who sought antibiotics to deal with the pressures of contemporary life:

GP2: There’s a group of especially young patients that want to heal immediately … It’s not unusual that a person comes and says “I have cold, I have fever, and in two days I have to go on a journey because it’s my holiday” or because I study in Belgium or because I am going to work, for instance. They are mostly young patients and with a lot of urgency to heal in [a] very short time. And in that case you say … “well, you must [wait] a week, I’m going to [prescribe] some things to make the symptoms not as important as you perceive them in this moment – with paracetamol, liquids and all that – but the cause of the infection will last a week.” And we’ve here in Catalonia, in Spain, we say “This is a cold and it will last a week, [but] with a doctor, [only] 7 days.”

He added that older people were familiar with old fashioned “grandma remedies” such as onion soup and were more receptive than young people when he suggested that the only treatment for a cold was bed, rest, and drinking a honey and lemon infusion.

Asked to provide examples of patients who had recently sought antibiotics, GP3 described how the previous day, a woman in her 40s had attended her clinic complaining of throat pain. GP3 had seen her before and knew she had also attended a private clinic. GP3 explained that this patient could not take time off work to rest. When told that she did not need antibiotics for a sore throat, the patient protested that she was always prescribed antibiotics and it was the only thing that would relieve the pain. GP3 said that in this instance, the patient was ultimately reassured by a negative strep-A test and the suggestion that she return in three days if her symptoms had not subsided.

Conversely, GP1 described how on numerous occasions, patients he knew well had asked for antibiotics, only to later admit that the medication was in fact for their pet dog. GP1 explained that all vets were private and there were significant expenses associated with animal medication, whereas prescription by a public sector GP entitles the patient to a 60% reduction on the face value of the medication. Patients would request an antibiotic prescription from him after realizing the name for the pet medication was the same as something they had previously been prescribed themselves and then seek the discount from their pharmacist.

While ingrained traditions based in Franco-era reliance on antibiotics were repeatedly invoked to explain why levels of antibiotic consumption remain relatively high in Catalonia, the above cases of antibiotic-seeking suggest that history and custom alone are insufficient to explain why GPs say they are so regularly asked for antibiotics. Expectations about receiving a prescription may reflect established conventions or be learned from private sector health care, from friends, or from the “hundreds of other ways in which [medical] knowledge is secreted in the social world” (Das 2015:221). Patient demand for antibiotics may have been cultivated over the years between physicians and Catalonia’s population, but it would also seem to reflect the realities of life and work in contemporary Catalonia.

### Sale without prescription

GP1: … if they go to the pharmacy to get antibiotics, they have to pay the full price. If you want a 60% discount, you have to go to the GP to persuade him to prescribe the antibiotic he/she have taken.

GP1 said that most of the direct requests for antibiotic prescriptions he receives are for medications that have already been consumed. He explained that patients frequently purchase the drug from a pharmacist who would tell them to return with a prescription to recover some of the cost of the medicine. Although illegal throughout Spain, the purchase of antibiotics from pharmacies without prescription has been widely reported (Llor and Cots 2009; Väänänen et al. 2006; Zapata-Cachafeiro et al. 2014, 2019). Our GP informants confirm that this practice continues to occur. Moreover, we were able to gain insights into over-the-counter purchasing of antibiotics from two people who had recently acquired antibiotics without a prescription.

The first of these, Re2, was a 32-year-old UK national who usually had access to private health care through his employer but was currently between jobs. Several days previously, he had begun to experience the symptoms of what, from experience, he suspected was tonsilitis. He had no Spanish social security number nor access to a GP and so tried to buy antibiotics at a pharmacy. The pharmacist was initially reluctant but acquiesced to Re2’s request after seeing white lumps on his tonsils and hearing that he did not have access to a doctor. Re2’s story may reflect the experience of those referred to by the Catalan media as “ex-pats” or “digital nomads” (ACN Barcelona 2022). Associated with processes of gentrification, these are non-nationals who live and work in Catalonia but receive salaries from abroad and are often not registered with local health and social security systems. Indeed, one of the few Spanish studies to explore the sale of antibiotics without prescription from the perspective of consumers was conducted among Finnish nationals living in Andalusia (Väänänen et al. 2006).

There is another group of people associated with the acquisition of antibiotics without prescription who, like the so called “ex-pats,” lack formal documentation and access to healthcare services. For instance, while GP2 also said over-the-counter acquisition was largely impossible, he later added that there were likely some pharmacists where it was still possible in the center of the city, indicating an area popular with tourists and home to many documented and undocumented migrants.^1^ Indeed, it was in this area that Re2 acquired antibiotics. GP2 thought these pharmacists were unscrupulous, but as Re2’s account indicates, such pharmacists may be persuaded by the knowledge that the customer in front of them cannot acquire treatment elsewhere. While this lies beyond the scope of this article, understanding antibiotics sold without prescription in Catalonia requires enquiry into the situated reasoning of local pharmacists who serve populations with high degrees of marginality and informality.

The second case is that of Re3, a 34-year-old Spanish national originally from Andalusia who worked at a language school in Barcelona. Re3 had twice tried to acquire antibiotics recently without a prescription. The first was while on holiday in Southern Spain. Her friend suggested that she ask for antibiotics in a pharmacy after she began to experience symptoms of a urinary tract infection (a recurrent problem for her). She was not successful, but later obtained a prescription from a GP in her nearby home city, where she was still registered with a primary care practice. When her symptoms reappeared several months later, Re3 saw a GP in Barcelona and was prescribed antibiotics. However, when she went to acquire the medication, the pharmacist said that the prescription was invalid due to an administrative error. She was told she could nevertheless purchase the medication and return later with a valid prescription to claim the 60% refund.

Notably, the ability to acquire antibiotics without prescription was not something afforded to everyone. Many residents with whom we spoke believed it was impossible to buy antibiotics save in specific circumstances – such as having a prescription with the wrong number, a handwritten note from a dentist, or being able to point to a history of prescribed antibiotics in one’s medical records.

## Conclusions: Prescription and use in ethnographic context

Antibiotics have been described as “quick fix infrastructures” (Willis and Chandler 2019) that compensate for inadequacies in health systems (Orzech and Nichter 2008). For patients too, antibiotics can seem to promise a “quick fix” to the health issues they face (Beckerleg et al. 1999). We see this in GP3’s account of the patient who could not take time off work to recover and in GP2’s reference to young patients who need to “heal immediately.” As the concept of a quick fix implies, however, antibiotics are often unsuitable, temporary remedies that cover for, rather than resolve, underlying problems such as deficits in health system funding and provisioning (Willis and Chandler 2019). As Rodrigues notes, when patients “decide to navigate the challenges inherent to any public healthcare service … they do expect medical solutions” (2020:9), but this can be in lieu of forms of care that are not available to them. GP1 said patients often sought reassurance that they did not have anything serious. While he and other GPs said it was preferable to explain to patients why they did not need antibiotics for viral infection, they also noted that this approach takes time. In the context of busy clinics, sometimes it was simply easier and quicker to write a prescription. Indeed, GP3’s account of her colleagues continuing to prescribe antibiotics even after a negative Strep-A test suggests that saving time may still be the overriding factor in the decision to prescribe antibiotics, even when physicians have access to adequate knowledge and technologies. In this sense, antibiotics effectively become a patch for systemic pressures associated with high patient numbers and limited human resources.

The programs of structural adjustment imposed on the Spanish economy in the years following the global financial crisis exacerbated longstanding problems associated with Spain’s aging population and historical deficits in its model of social care provision (Campillo-Poza 2018; McVeigh 2005; Miguélez and Recio 2010; Pérez-Orozco 2006). In primary care, pressures increased as a result of the retrenchment of other care services (Cervero-Liceras et al. 2015; Departament de Salut 2021) as well as rising levels of morbidity associated with the effects of the reforms, most notably in mental health (Gili et al. 2013). But as recent ethnographic studies have illustrated (Knight and Steward 2016; Narotzky 2020) among the most insidious and enduring effects of austerity in Southern Europe has been the undermining of everyday relations of social reproduction, that is, “the material social practices through which people reproduce themselves on a daily and generational basis and through which … social relations … are renewed” (Katz 2001:709). At the same time as welfare retrenchment increased the burden of care on individuals and household, employment became more precarious and real wages fell, increasing the amount of paid work needed to support a family (Buendía and Molero-Simarro 2018). The result is what some have described as a crisis of care (Campillo-Poza 2018), a multi-directional squeeze on time and social capacities that exacerbates existing socio-economic inequalities.

While our focus in this article has been on the clinic rather than the community, we nevertheless find elements of this care crisis reflected in one GP’s view that working age patients are anxious to get better fast to avoid taking time off work. Re2’s attempts to acquire antibiotics without prescription likewise speak to links between health issues, work and access to care. In follow-up interviews, Re3 narrated an incident that had taken place in her school a few months earlier, during which her boss had harangued her for trying to leave work after she became pallid and faint during a class. The situation was resolved after Re3 agreed to go to a pharmacy and send her boss a blood pressure reading via WhatsApp. Re3 explained that she had previously lost her job at the same school during the financial crisis. After a period living abroad, she returned to the school on the promise of a new permanent job. However, student numbers had recently been falling again and weekly teaching hours had been reduced. According to Re3, everyone working in the school understood that taking time off work could jeopardize continued employment. Likewise, GP3 commented that one of the changes occasioned by the financial crisis was that unemployment and job insecurity became so severe that many of her patients no longer wanted sicknotes to take time off work but only medicine to get better quicker. It is in this economic context that antibiotics take on significance as a possible substitute for managing illness.

In global health discourses on AMR, antibiotic prescription and use tend to be framed as matters of individual behavior, leading to policy interventions that are targeted at changing the attitudes and beliefs of patients and prescribers (Lambert et al. 2019). Our clinical informants suggested that their colleagues’ and patients’ attitudes had been changing in recent years, reflecting the rise of public health messaging and professional education on antibiotic stewardship. However, their accounts also illustrate the limitations of these efforts directed at individual behavior change: while they may succeed in changing attitudes, they do not alter the material circumstances that generate the (perceived) need for antibiotics.

In this article, we have sought to move beyond individual behavior by situating antibiotic prescription and use in Catalonia within its historical and contemporary socio-economic context. Our ethnographic approach entailed layering historical, resident, patient, and prescriber perspectives and revealed antibiotic prescription and use to be connected to a range of structural economic concerns. Spain’s position as one of the largest per capita consumers of antibiotics in Europe undoubtedly reflects the country’s historic overreliance on antibiotics consequent on various inadequacies in public health and welfare provision, as many informants confirmed. But this leaves unanswered the question of why such practices are still widespread. Our research suggests that traditional practices of antibiotic prescription and consumption may be repurposed to deal with a range of contemporary pressures associated with the intensification of work and deficits in the provision of care. Interrogating the relationship between prescription, use and these broader political economic concerns sheds light on the reasons that practices apparently rooted in the Franco era continue to be reproduced some 40 years after the end of the regime.

## Note

1. Undocumented migrants were excluded from public health care coverage by reforms introduced following the financial crisis (Cervero-Liceras et al. 2015).
